# Experimental Research on the Bond Performance between SMAF-ECC Composites and Steel Bar

**DOI:** 10.3390/ma16145037

**Published:** 2023-07-17

**Authors:** Zhao Yang, Shuai Li, Feng Gao, Rui Wang

**Affiliations:** 1 School of Urban Construction, Wuhan University of Science and Technology, Wuhan 430065, China; ls1280068396@163.com (S.L.); 18684718538@163.com (F.G.); wr15972022840@163.com (R.W.); 2 Hubei Provincial Engineering Research Center of Urban Regeneration, Wuhan University of Science and Technology, Wuhan 430065, China

**Keywords:** ECC, shape memory alloy fiber (SMAF), bond performance, tensile test, slip

## Abstract

Combining Engineered Cementitious Composites (ECC) with shape memory alloy (SMA) fibers can form SMA fiber reinforced ECC (SMAF-ECC) that has excellent deformation recovery and energy dissipation capabilities. Substituting some of the tensioned concrete with this new composite material, along with steel bars, is expected to significantly improve the seismic energy dissipation and self-recovery capabilities of traditional reinforced concrete components. However, a reliable bond between steel bars and SMAF-ECC is critical to ensure their synergistic performance. In this paper, the failure mode and bond strength of steel bars and SMAF-ECC were studied through direct tensile tests, and the influence factors such as steel bar diameter, bond length, and SMAF volume fraction were analyzed. A bond-slip constitutive model for steel bars and SMAF-ECC was proposed. The results show that the failure mode of the tensile test specimens is mainly steel bar pull-out failure; the incorporation of SMAF significantly enhances the bond strength between the steel bar and matrix; increasing the steel bar diameter and bond length both lead to a decrease in bond strength while increasing the SMAF volume fraction can significantly increase the bond strength. Among them, the specimen with a steel bar diameter of 12 mm, bond length of 70 mm, and SMAF volume fraction of 0.5% has the largest increase in bond strength, reaching 52.96%. The proposed improved bond-slip constitutive model is in good agreement with the bond-slip curve obtained in the experiments, with a determination coefficient of 0.99. The research results of this paper provide an important theoretical basis for promoting the engineering application of SMAF-ECC materials.

## 1. Introduction

Extensive research has been conducted on the bond characteristics between steel bars and concrete, highlighting the crucial role of a strong bond in harnessing their synergistic potential and guaranteeing the optimal performance of reinforced concrete structures [1,2]. Concrete, an indispensable construction material in the field of civil engineering, has garnered widespread adoption owing to its versatile properties and manifold applications [3]. Due to its brittle nature and low tensile strength, it is prone to cracking when subjected to tensile forces, which seriously affects its bond performance with steel bars [4]. Therefore, replacing concrete with a ductile material with good tensile properties has become an important development direction to prevent crack propagation and enhance the bond capacity between steel reinforcement bars and the surrounding matrix [5].

Engineered Cementitious Composites (ECC) are high ductility cement-based composite materials with excellent tensile properties and can reach a maximum tensile strain of over 3% [6,7,8,9]. By using short fibers to bridge cracks, ECC can control crack width within a small range. Related studies have shown that when the stretch strain of the matrix exceeds 1%, the width of cracks in ECC remains stable at around 60 μm, indicating good crack resistance [10]. Shape memory alloy (SMA) is a novel material exhibiting exceptional shape memory effect or superelasticity, along with inherent capabilities of self-sensing, self-diagnosis, and self-adaptation [11]. The superelasticity of SMA is stimulated by stress and can exhibit hysteresis loop energy dissipation characteristics under cyclic loading, and can recover deformation after unloading [12]. SMA-ECC composites have broad prospects for application in civil engineering, as some research has demonstrated that SMA combined with ECC can provide excellent mechanical properties for structures. Qian et al. [13] reinforced concrete beams with SMA-ECC, and the experiment showed that the SMA-ECC improved the self-resetting, malleability, and ability to absorb the energy of the beams. Hung [14] studied the bending performance of SMA-ECC and ECC beams with steel bars, and the experiment exhibited that SMA-ECC improved the maximum deflection of the beam and demonstrated good energy dissipation and self-repairing properties.

However, most of the current research on SMA materials uses rods or wires, which have problems such as difficult processing, the need for specific connection devices, many defects, and high costs, which are not conducive to the promotion and application of SMA. Therefore, some scholars have begun to use shape memory alloy fibers (SMAFs) instead of SMA rods or wires. This type of fiber has the advantages of easy processing, few defects, no need for connection devices, and low cost. Choi et al. [15,16] found that curly SMA fibers in mortar have a strong anchoring force by pull-out experiments of curly SMA fibers in mortar. Wang et al. [17] conducted experimental research on the tensile behavior of knotted SMA fibers reinforced composite materials, and they found that knotted SMAFs were more effective in closing the joint than straight SMAFs and could provide sufficient recovery force. Luo [18] controlled the expansion of V-shaped notches in the matrix by using knotted shape memory alloy fibers, and the results showed that knotted shape memory alloy fibers could effectively control the expansion of the notch and improve the tensile strength of the composite material. Shajil et al. [19] conducted three-point loading tests on prismatic and specially designed beam-column connection prototypes with steel fibers and shape memory alloy fibers. The results showed that the composite material containing shape memory alloy fibers had excellent ductility and self-centering capability.

Combining SMAF with ECC can fully utilize the advantages of both materials, forming a new type of composite material called SMAF-ECC with high ductility, energy dissipation, and self-recovery properties [20,21]. In Reference [22], cyclic tensile tests were conducted on an ECC matrix containing SMA fibers, and the results showed that knotted end can ensure a sufficient anchoring ability for SMAFs, and the superelasticity of the SMA material can be effectively stimulated. In addition, the authors [23] conducted bending tests on the beams with predetermined notches, and the results demonstrated that SMAF exhibited notable abilities in dissipating energy and recovering deformation, significantly improving the self-centering performance of the beams.

Although the SMA-ECC material demonstrated outstanding mechanical characteristics., the relatively high cost of this new material compared to traditional reinforced concrete and its limited tensile strength make it more feasible to partially replace the concrete in the region subjected to tensile forces and co-bear the load with reinforcing materials such as steel bars. However, the establishment of strong bonding between SMAF-ECC and rebars is crucial to ensure their synergistic effect. Although there have been many researches on the bonding performance between rebars and ECC materials [24,25,26,27], the significant differences in material composition and mechanical properties between SMAF-ECC and conventional ECC indicate that the bond interface between steel bars and SMAF-ECC will exhibit distinct characteristics in terms of failure mode, bond strength, and bond-slip behavior. Therefore, it is necessary to conduct specific research on the bond mechanics between steel bars and SMAF-ECC. Therefore, the paper investigated the bond mechanics between steel bars and SMAF-ECC through tensile tests, analyzed the failure mode and bond-slip mechanism of the test specimens, and compared the influence factors such as rebar diameter, bond length, and SMAF content on the bond mechanics. The findings of this investigation establish a solid basis for the utilization and implementation of SMAF-ECC in the construction of reinforced concrete systems.

## 2. Test Overview

### 2.1. Test Material

#### 2.1.1. Engineered Cementitious Composites (ECC)

The composition ratio of the ECC material [28] is detailed in Table 1. The cement employed was classified as type II Portland cement, exhibiting a compressive strength of 42.5 MPa. The fly ash selected possessed a density of 2.42 g/cm^3^ and a fineness within the range of 11–12%. The sand utilized comprised white crystalline quartz sand with a fineness of 140 mesh. The PVA fiber implemented originated from Japan and was characterized by its high-strength and high-modulus properties, featuring a diameter measuring 31 μm and a length of 9 mm. The PVA fiber exhibited an impressive tensile strength of 1600 MPa and an elastic modulus of 42 GPa. The water reducer used was a 540 P polycarboxylate high-performance water reducer produced by Wuhan huaxuan high-tech Co., Ltd. (Wuhan, China).

**Figure 1:**
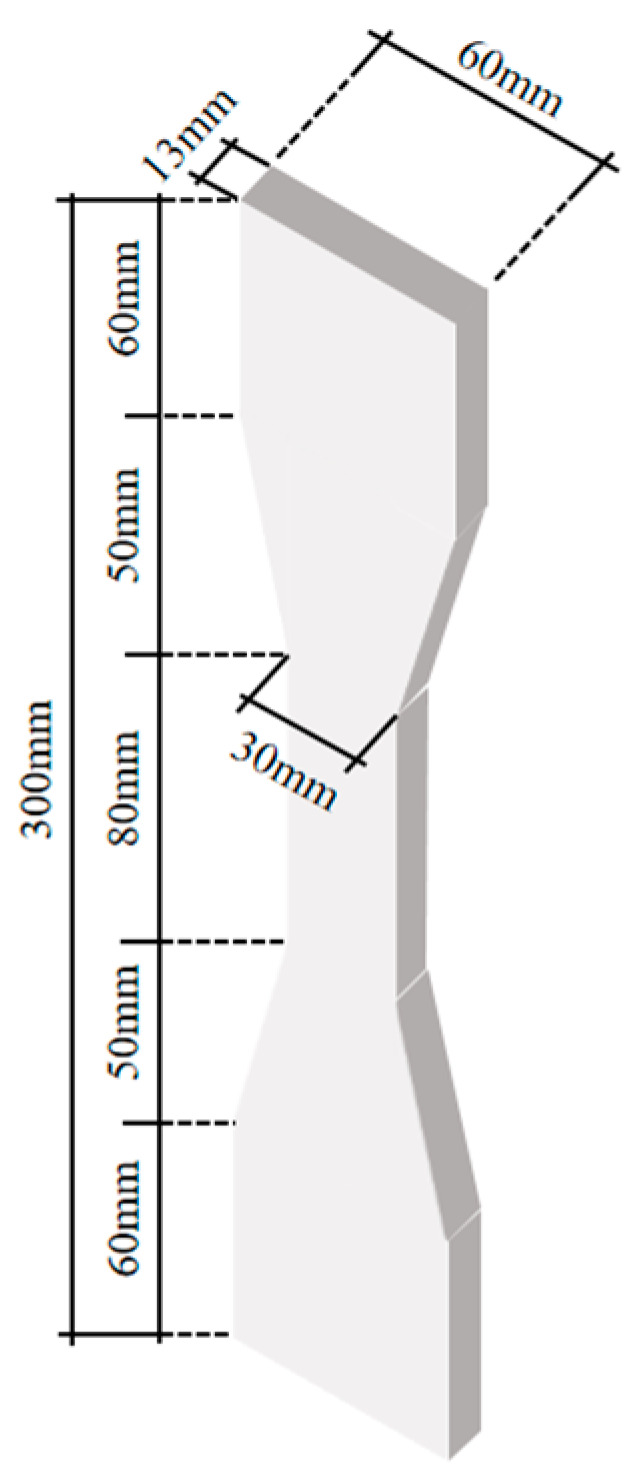
Dimensions of the ECC specimen.

In accordance with Chinese code JC/T 2461-2018, which pertains to the standardized testing methodology for evaluating the mechanical properties of ductile fiber-reinforced cementitious composites [29], the dog-bone-shaped specimens were fabricated using the composition ratio of ECC detailed in Table 1, and cured under standard conditions for 28 days. The precise dimensions of the ECC specimens are visually represented in Figure 1.

**Figure 2:**
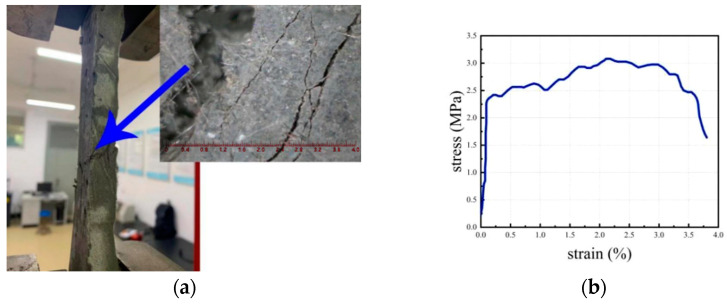
Test results of the ECC specimen. (**a**) Crack; (**b**) Stress-strain curve.

The tensile testing of the ECC specimens was performed using a WD-P6 universal testing machine manufactured by Jinan Puye, a Chinese company (Jinan, China). The testing procedure employed the displacement control mode, maintaining a loading rate of 0.2 mm/min. The loading process was halted upon the initiation of the primary crack. Notably, during the tensile testing, the specimens exhibited distinctive characteristics of multiple cracking, as visually depicted in Figure 2a. Furthermore, the ECC material demonstrated evident strain hardening behavior, with an ultimate tensile strain surpassing 3%, as demonstrated in Figure 2b.

#### 2.1.2. Shape Memory Alloy (SMA)

**Figure 3:**
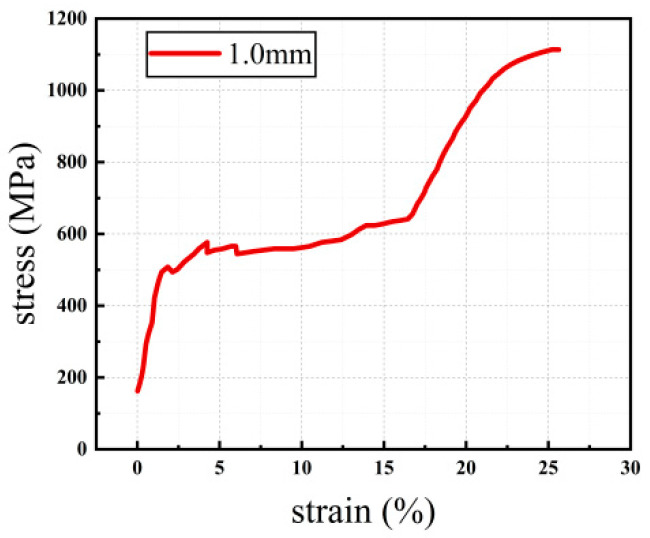
Tensile stress-strain curves of shape memory alloys.

The experiment used a 1.0 mm diameter SMA alloy wire, mainly composed of 55.86% nickel and 44.14% titanium, with an austenitic structure in a standard room environment. According to Reference [30], direct tensile tests were conducted on the SMA wires using a TH-8201A type 20 kN universal material testing machine produced by Suzhou TOP company from China (Suzhou, China). The specimens were set to a gauge length of 100 mm, and the tensile tests were conducted employing a loading procedure controlled by displacement at a rate of 2 mm/min. The obtained stress-strain curve from the tensile test is depicted in Figure 3, whereas the relevant mechanical characteristics are consolidated and presented in Table 2.

According to Figure 3 and Table 2, the stress-strain curve of SMA shows a distinct phase transition plateau, indicating excellent superelasticity of the SMA material at room temperature. The starting stress and strain of the phase transition stress plateau during tension are 441.86 MPa and 1.55%, respectively, and the ending stress and strain of the phase transition stress plateau are 589.63 MPa and 16.29%, respectively. The ultimate tensile strength reaches 1111.01 MPa, and the ultimate strain is 25.53%.

#### 2.1.3. Steel Bar

In this experiment, three diameters (12 mm, 14 mm, and 16 mm) of HRB400 ribbed steel bars were used. Three specimens were selected for each diameter to conduct axial tensile experiments, and the mechanical characteristics of the rebars are presented in Table 3.

### 2.2. Design of Tensile Test

**Figure 4:**
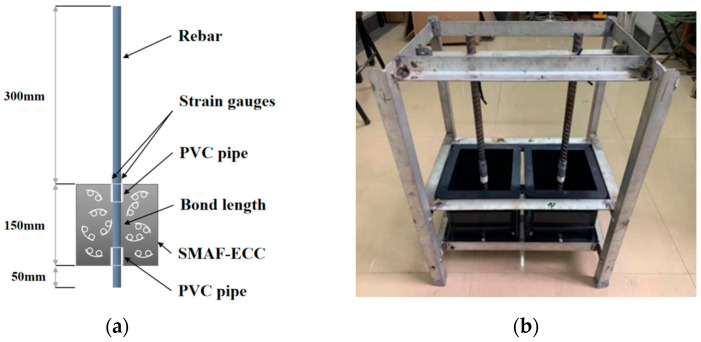
Design Drawing of Tensile Specimen. (**a**) Specimen dimensions; (**b**) Specimen preparation.

**Figure 5:**
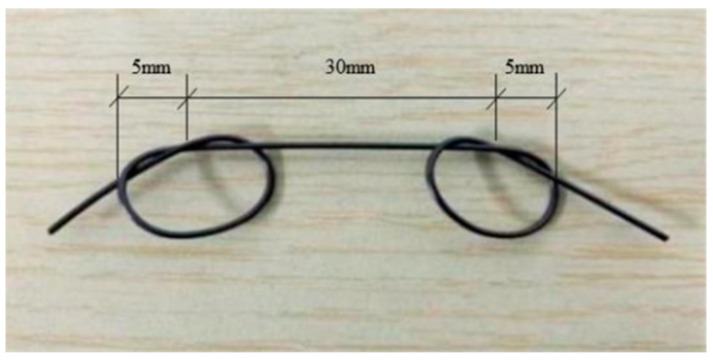
SMA knotted at the end.

In accordance with the research objectives of this experiment, a central tensile test was conducted to study the bonding behavior between the rebars and SMAF-ECC, and to compare the three influencing factors: steel bar diameter, bond length, and SMAF volume fraction. Based on GB50152-92 [31] and JGJ55-2011 [32], 11 groups of a total of 33 tensile specimens were designed, as listed in Table 4. The tensile specimen dimensions are represented in Figure 4a, and the ECC matrix is a cube with a side length of 150 mm. A self-made iron frame and mold shown in Figure 4b were used to cast the specimens. To mitigate stress concentration at the two ends of the bonded section of the matrix and the steel bar, a PVC pipe was utilized to create a physical separation between the steel bar and the matrix, establishing an unbonded region. The steel bar was threaded through the perforated mold, and the loading end was fixed on the iron frame to avoid eccentricity. Then, the SMA fibers with knotting end heads were evenly placed in the mold while pouring PVA-ECC, and the samples underwent compaction after the application of vibration and had their molding removed after 24 h of storage at room temperature. Finally, the samples underwent a curing process for a duration of 28 days in a controlled environment with a temperature maintained at 20 ± 2 °C and a humidity level exceeding 95% prior to testing. Knotting end heads were set for the SMA fibers in the experiment, with a spacing of 30 mm and a diameter of 10 mm (Figure 5).

### 2.3. Test Loading and Measuring Devices

**Figure 6:**
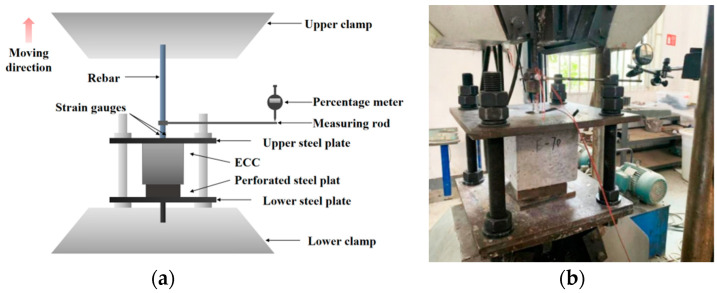
The setup of the test apparatus for applying loads and measuring parameters. (**a**) Schematic diagram; (**b**) On-site photograph.

The experimental arrangement for the tensile test is illustrated in Figure 6. The WAW-1000 microcomputer-controlled electro-hydraulic servo testing system, manufactured by Jinan Test Gold Group Company in China, was employed to apply the tensile load to the specimen. The loading magnitude was controlled by displacement, and the loading speed was set at 0.2 mm/min. The load data are automatically collected by the universal testing machine. The loading is terminated when the failure of the matrix, steel bar fracture, or pull-out occurs. Strain gauges (BE120-3AA) are symmetrically arranged on the steel bar loading end to capture the deformation of the steel bar., and the strain gauge data are recorded by the BZ2205C program-controlled static resistance strain meter. The movement at the loading end of the rebar is measured using a dial gauge.

**Figure 7:**
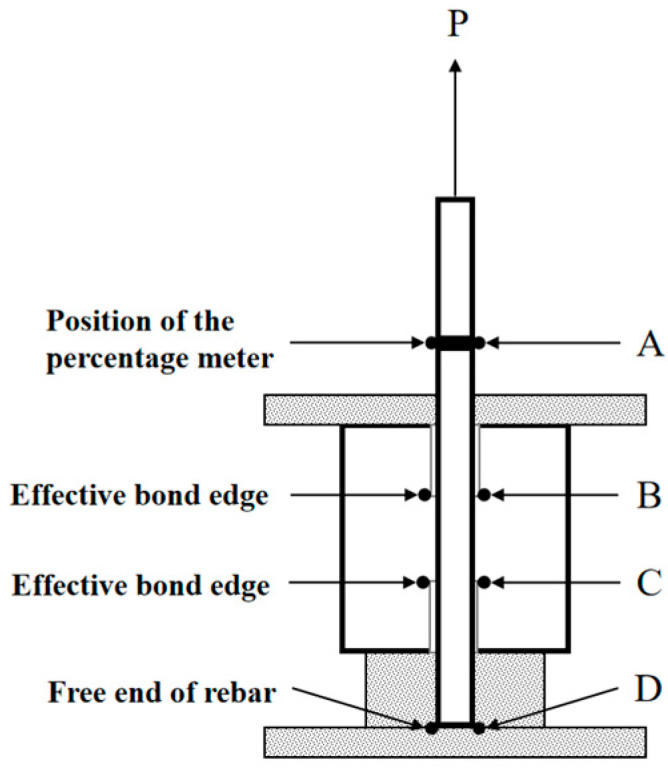
Percentage table layout schematic.

In Figure 7, the BC segment represents the actual bonded section between the steel bar and the matrix; the CD segment is the free segment where the steel bar undergoes no deformation during loading; the AB segment is the section that directly bears the tensile load. Point A is the measuring point of the dial gauge, and the strain gauge is placed at the midpoint of the AB segment to measure the average strain ε of the steel bar.

As shown in Figure 7, the actual slip of the loading end during the loading process is obtained by Formulas (1) and (2).

(1) $$S_{f}=S_{m}-S_{d}$$

(2) $$S_{d}=\varepsilon \times L_{AB}$$

where: $S_{f}$—Actual slip at the loading end, in units of mm; $S_{m}$—Measured displacement at the loading end, in units of mm; $S_{d}$—The elastic deformation of the steel bar in section AB, in units of mm; *L_AB_*—AB section steel bar length, in units of mm.

## 3. Test Results and Analysis

### 3.1. Test Material

**Figure 8:**
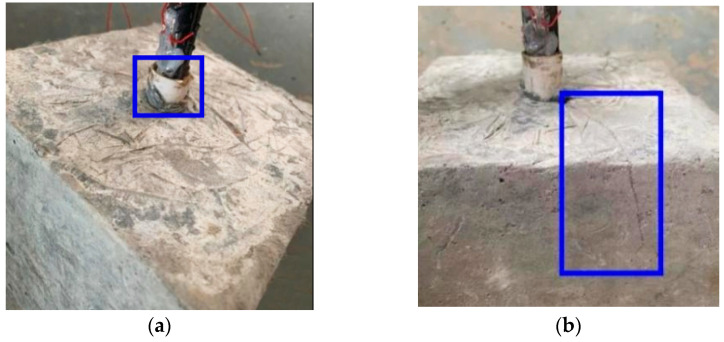
Failure mode. (**a**) Pull-out failure; (**b**) Pull-out splitting failure.

The outcomes of the tensile tests conducted on each specimen are presented in Table 5. The observed modes of failure in the specimens primarily involved pull-out and pull-out splitting, as depicted in Figure 8. Among these, the majority of specimens demonstrated pull-out failures, with only the specimen featuring a steel bar diameter of 16 mm exhibiting a pull-out splitting failure. The experimental findings indicate that in cases of pull-out failure, the steel bar was extracted from the matrix without significant damage. However, in the case of a pull-out splitting failure, the cracks were not obvious before the specimens reached the ultimate load during the test, and the cracks appeared simultaneously with the ultimate load. After that, the cracks continued to develop and increase, while the bond strength of the substrate decreased. It is worth noting that the crack development of the specimens all occurred from the bond area to the non-bond areas on both sides, but there was no penetrating crack at the point of failure, this is different from the experimental results of the literature [24], which is mainly due to the good tensile capacity of the SMAF-ECC substrate [20,21].

### 3.2. Bond Strength

#### 3.2.1. Test Results

According to Reference [33], by simplifying the bonded reinforcement in the test to a cylindrical body, the average bond stress between the steel reinforcement and the SMAF-ECC matrix interface can be obtained using Equation (3):

(3) $$\tau =\frac{P}{\pi dl_{a}}$$

where τ represents the average bond stress in MPa; *P* denotes the test tensile load in N, d represents the diameter of the steel bar in mm, and $l_{a}$ represents the bond length in mm. The peak bond stress *τ_u_* corresponds to the peak load Pu. The average bond strength of the test specimens calculated using Equation (3) is presented in Table 5.

Through comparison of the experimental data in Table 5, it can be concluded that under the same conditions of steel bar diameter and bond length, the specimens containing SMAF show a significant improvement in bond strength compared to those with 0% volume fraction of SMAF. This is attributed to the fact that the interlocking SMA particles can be effectively anchored to the ECC matrix, and can play a bridging role during the loading process, thereby controlling crack propagation and increasing the tensile strength of the ECC matrix, and ultimately enhancing the bond strength between the matrix and the steel bar [34].

#### 3.2.2. Influencing Factors

##### Diameter of the Steel Bar

**Figure 9:**
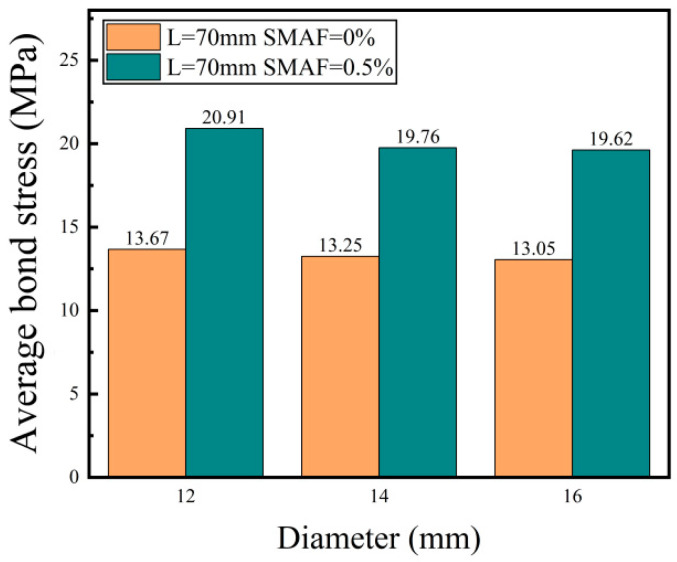
Effect of different diameters on bond strength.

In this study, two groups of test specimens were chosen, featuring a bond length of 70 mm and SMAF volume fractions of 0% and 0.5%, respectively. The aim was to examine the impact of varying steel bar diameters on the bond strength, utilizing the bond strength data presented in Table 5 as a basis for analysis. As shown in Figure 9, the bond strength between steel bars and SMAF-ECC slightly decreases with an increase in steel bar diameter. When the SMAF volume fraction is 0%, compared to the specimen with a steel bar diameter of 12 mm, the bond strength of specimens with steel bar diameters of 14 mm and 16 mm decreased by 3.1% and 4.5%, respectively. When considering a SMAF volume fraction of 0.5%, a decrease of 5.5% and 6.2% in bond strength was observed in specimens with steel bar diameters of 14 mm and 16 mm, respectively, in comparison to the specimen with a 12 mm steel bar diameter. This occurrence can be primarily attributed to the utilization of ribbed steel bars in the experiment, where the bond force between the ribbed steel bars and the matrix relies on the mechanical interlocking force generated by the transverse ribs of the steel bar engaging with the matrix ribs. The main steel bar geometric parameters that affect the interlocking force are rib height and rib spacing. For steel bars with the same diameter, the higher the transverse ribs and the smaller the rib spacing, the better the interlocking effect. In order to eliminate the influence of different steel bar diameters, the concept of relative rib height (rib height/diameter) and relative rib spacing (rib spacing/diameter) is introduced [35]. With an increase in steel bar diameter, the relative rib height of the steel bar gradually decreases, and the number of transverse ribs contained in the steel bar within the same bond length range also decreases. Therefore, under the condition of other influencing factors being the same, as the steel bar diameter increases, the rib height relatively decreases, the rib spacing relatively increases, and the mechanical interlocking force decreases, leading to a bond strength decrease [36].

##### Bond Length

**Figure 10:**
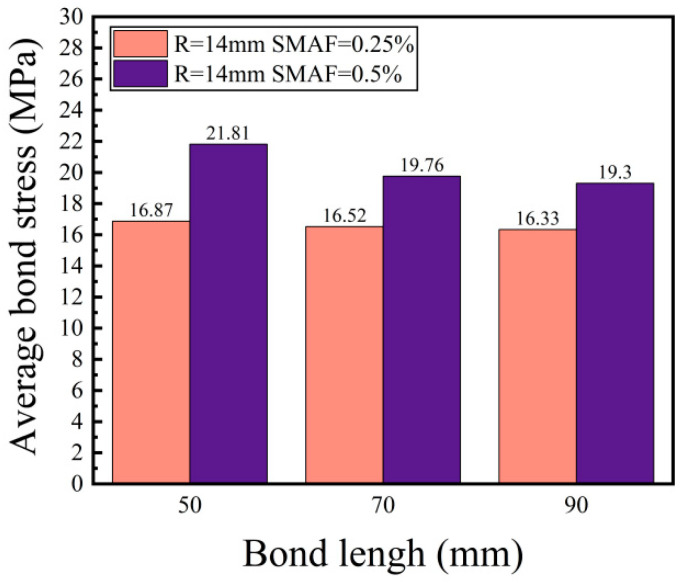
Effect of different bond lengths on bond strength.

Two groups of specimens were chosen to analyze the influence of different bond lengths on the bonding strength between 14 mm diameter steel bars and SMAF-ECC, with SMAF volume fractions of 0.25% and 0.5%, respectively. It can be seen in Figure 10, the bond strength between the steel and SMAF-ECC decreases to varying degrees with an increase in bond length this is consistent with the findings of the literature [24] and the literature [26], but differs from the findings of the literature [27] due to the short range of bond lengths studied in the literature [27] and the increase in bond strength with increasing bond length, which is different from the results of this paper. When the volume fraction of SMAF is 0.25%, compared to the specimens with a bond length of 50 mm, the bond strength of the specimens with bond lengths of 70 mm and 90 mm decreased by 2.1% and 3.2%, respectively. When the volume fraction of SMAF is 0.5%, compared to the specimens with a bond length of 50 mm, the bond strength of the specimens with bond lengths of 70 mm and 90 mm decreased by 9.4% and 11.5%, respectively.

Although an increase in bond length results in an increase in the bond area between the steel and the matrix, and hence an increase in mechanical anchoring force, research has shown that there must be an effective bonding length between the rebar and the matrix, and continuing to increase the bond length beyond this effective length cannot lead to a significant increase in bond strength [37]. When the rate of increase in pulling force is less than that of the increase in bond area, the average bond stress decreases. Moreover, during the pulling process, the bonding stress between the rebar and matrix is unevenly distributed along the bond length, and this non-uniform stress distribution becomes more pronounced as the bond length increases, ultimately resulting in a reduction in average bond stress [38]. Additionally, as the bond length is extended, the peak load experienced by the specimen increases, along with an increase in tensile strain at the loading end of the steel. This, in turn, triggers radial shrinkage of the steel due to Poisson’s effect, leading to a decrease in the constraint force exerted by the matrix on the steel. Consequently, the bond strength is adversely affected.

##### Volume Fraction of SMAF

**Figure 11:**
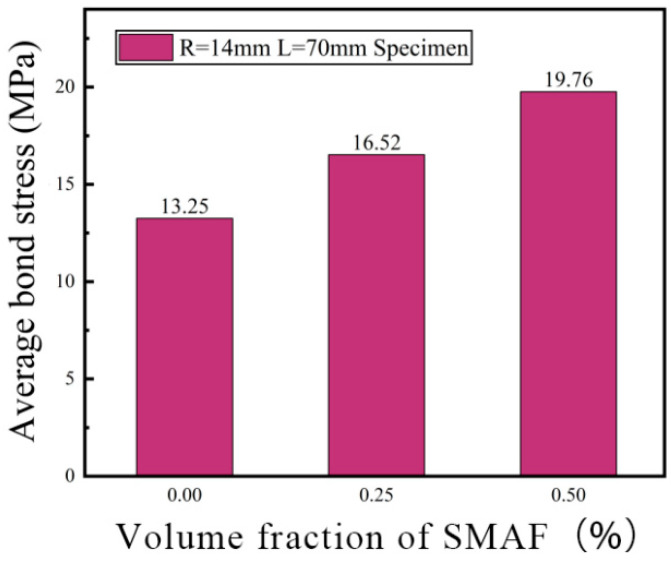
Effect of different volume fractions of SMAF on bond strength.

Samples featuring a steel bar diameter of 14 mm and a bond length of 70 mm were chosen to examine the impact of various SMAF volume fractions on the bond strength between steel bars and SMAF-ECC. As depicted in Figure 11, the bond strength between steel bars and SMAF-ECC demonstrates an upward trend with an increase in the SMAF volume fraction. For specimens with a steel bar diameter of 14 mm and a bond length of 70 mm, the bond strength of samples with SMAF volume fractions of 0.25% and 0.5% exhibited enhancements of 24.68% and 49.13%, respectively, compared to specimens with a SMAF volume fraction of 0%. These results indicate that when cracks propagate inside the specimen, the crack tip will be hindered by the SMA fibers when it extends to the SMA fibers, resulting in a reduction in the crack propagation rate. After cracking, the SMA fibers can effectively play a bridging role together with PVA fibers to suppress the opening of cracks in the matrix, thereby improving the shear strength of the matrix and the mechanical interlocking force between the matrix and steel bar ribs. The higher the SMA fiber volume fraction, the more SMA fibers can play a bridging role, resulting in a higher bond strength, which is consistent with the law in the literature [20]. At the same time, during the tensile process of the steel bar, the steel bar ribs within the bond length will compress the surrounding matrix, resulting in diagonal and radial cracks in the matrix. The addition of SMA fibers will increase the tensile strength of the matrix [39], improve the mechanical anchoring force between the matrix and steel bar, and thus improve the bond strength of the specimen.

### 3.3. Bond-Slip Relationship

**Figure 12:**
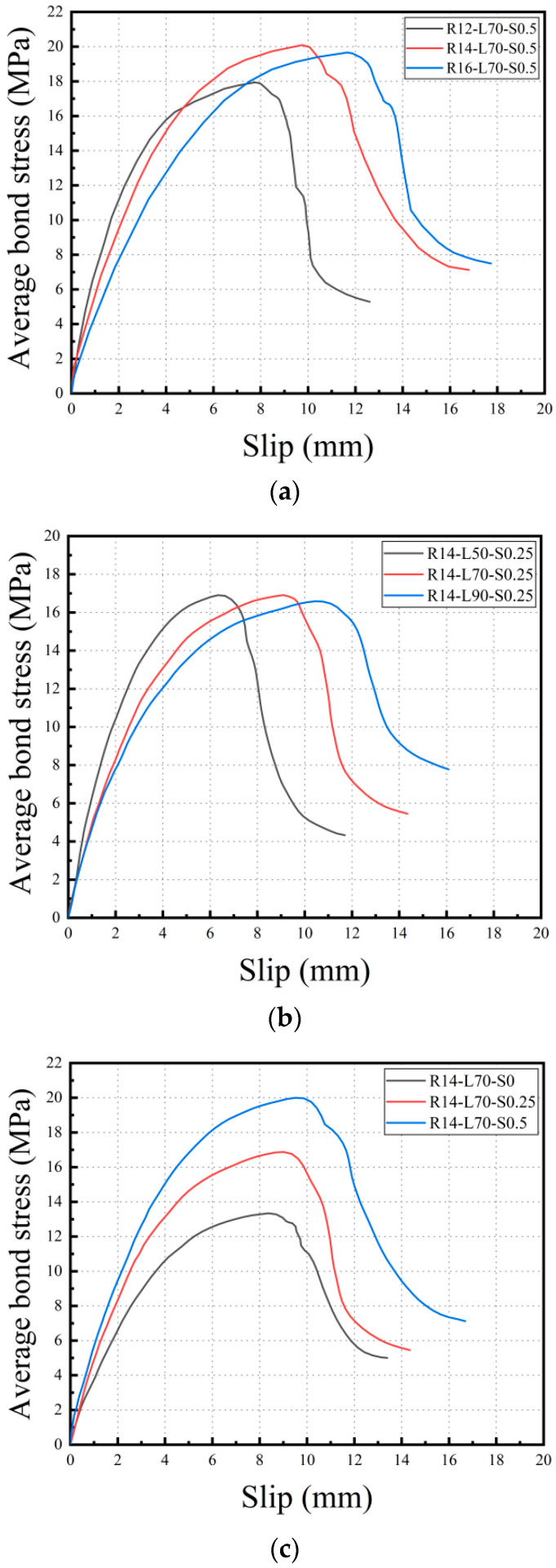
Bond-slip curve. (**a**) Comparison of different reinforcement diameters at s = 0.5; (**b**) Comparison of different bond lengths at s = 0.25; (**c**) Comparison of different SMAF volume fractions at R = 14 mm and L = 70 mm.

By utilizing Formulas (1)–(3), the actual slip of the steel bars and the average bond stress can be computed based on the recorded displacement and corresponding load data. Plotting the average bond stress against the actual slip enables the construction of the bond-slip curve. This curve facilitates the analysis of the bond-slip behavior of the specimen under various influential factors. Figure 12 illustrates that the bond-slip process of the specimen can be categorized into three distinct stages.

(1) The initial cracking stage: the bond-slip curve appears as a straight line. At this stage, the bond between the steel bar and the matrix is mainly controlled by chemical adhesion forces. Despite the fact that several micro-cracks propagate from the top of the protruding ribs, most of the cracks remain in their original or undeveloped state due to the low level of the applied load. The action of the SMA and PVA fibers at this stage has not been activated.
(2) The stable crack extension stage: the bond-slip curve enters a non-linear rising phase with a decreasing slope. In this stage, force transfer is primarily controlled by the mechanical interlocking force between the rib of the reinforcing steel and the matrix. As the applied load increases, the micro-cracks in the specimen begin to expand continuously at the matrix and rib of the reinforcing steel, leading to the development of multiple fine cracks, thus causing a continuous softening of the bond reaction. During this period, the cracks in the specimen have a certain width, and the bridging effect of SMA and PVA fibers in the matrix helps to suppress crack propagation, allowing the specimen to achieve a higher maximum bond strength.
(3) The unstable crack propagation stage: the bond-slip curve enters the descending phase. Micro-cracks in the specimen gradually develop and accumulate, forming large cracks, and the speed of crack propagation becomes unstable. The load-carrying capacity of the specimen continuously decreases until shear failure occurs.

### 3.4. Bond-Slip Constitutive Model

**Figure 13:**
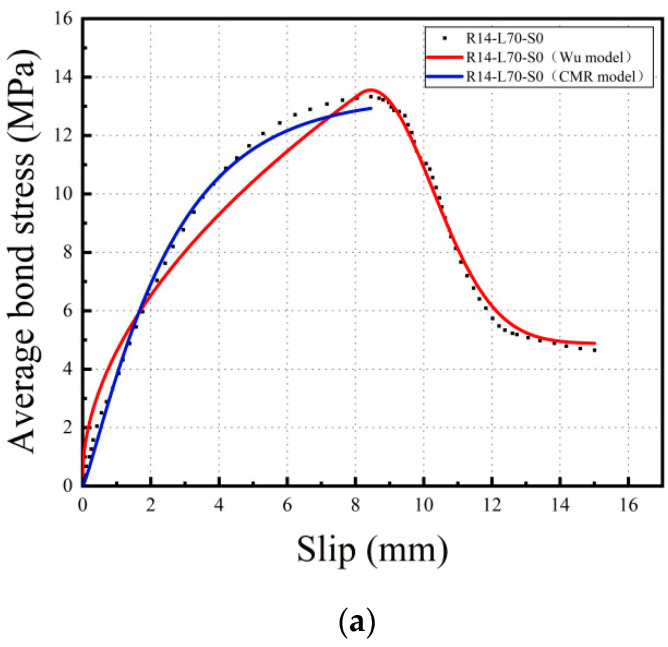

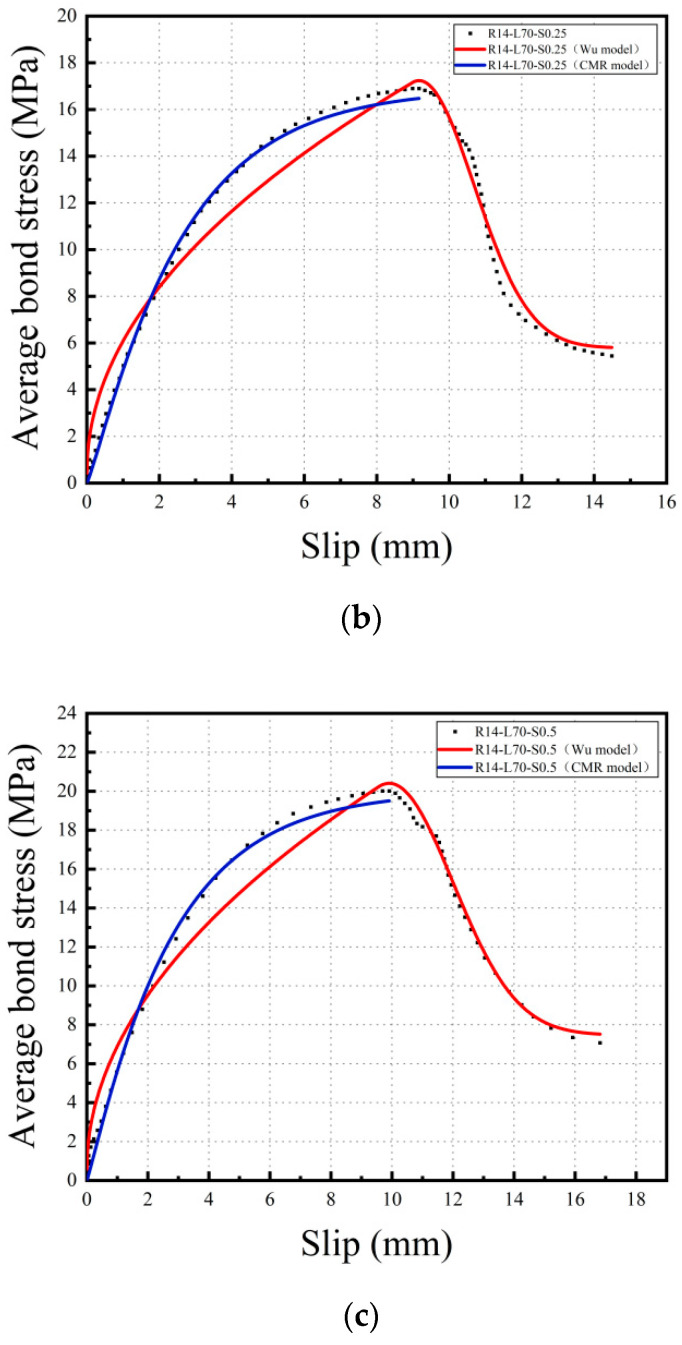
Comparison of the experimental curve and fitting model curve. (**a**) R14-L70-S0 comparison; (**b**) R14-L70-S0.25 comparison; (**c**) R14-L70-S0.5 comparison.

The bond-slip constitutive model is a crucial tool for describing the bond behavior between steel reinforcement and the matrix. It plays a significant role in the theoretical analysis and numerical simulation of material mechanical properties. In this study, the bond-slip curve obtained from the current experiment was analyzed and fitted using the bond-slip constitutive model proposed by Wu [40] and the CMR model [41]. The mathematical expressions for the Wu model and the CMR model are provided in Equations (4) and (5) respectively, and the fitting parameters are listed in Table 6. Figure 13 illustrates the comparison between the fitted curve and the experimental curve.

Wu model:

(4) $$\tau =\left\{ \begin{array}{ll} \tau_{u}{\left(s/s_{u}\right)}^{\alpha } & \left(0\leq s\leq s_{u}\right)\\ \tau_{u}\left\{\tau_{0}/\tau_{u}+(1-\tau_{0}/\tau_{u})\exp\left[\beta {\left(s/s_{u}-1\right)}^{2}\right]\right\} & \left(s_{u}<s\leq s_{0}\right) \end{array}\right.$$

CMR model:

(5) $$\tau =\tau_{u}{\left[1-\exp\left(-s/s_{r}\right)\right]}^{p}$$

where τ represents the average bond stress in MPa, s represents the slip value in mm, τ*_u_* represents the maximum bond strength in MPa, *s_u_* represents the corresponding slip value when reaching the maximum bond strength in mm, and τ_0_ represents the bond strength corresponding to the maximum slip value in MPa. The parameters *α*, *β*, *S_r_*, and *p* are determined through regression analysis.

Based on Table 6 and Figure 13, it can be observed that the CMR model has a better fitting effect than the Wu model before the average bond stress reaches the bond strength, with R^2^ values of approximately 0.99. However, after the average bond stress reaches the bond strength, the CMR model fails to continue fitting the curve, while the Wu model exhibits higher accuracy with R^2^ values of approximately 0.96–0.97.

**Figure 14:**
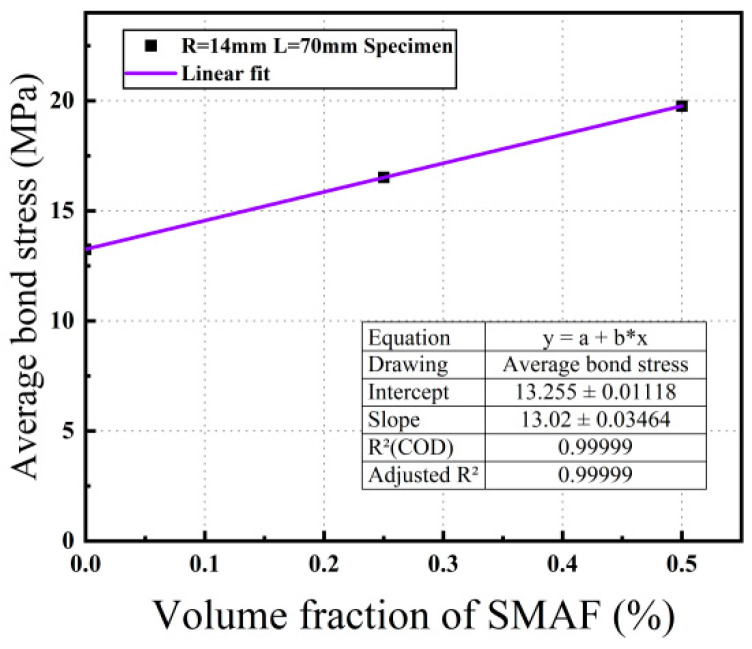
Fit curve.

Based on the results of previous experimental studies, the bond strength is affected by multiple factors, among which the volume fraction of SMAF is a significant factor that has not been considered in existing theoretical models, but its impact on bond strength is quite apparent. Therefore, this paper uses Origin 2021 software to perform linear regression analysis on the data in Figure 11, as shown in Figure 14, to obtain the relationship expression between the volume fraction of SMAF x and the bond strength influence coefficient $\psi_{\left(x\right)}$, as given in Equation (6).

(6) $$\psi_{\left(x\right)}=13.25+13.02x$$

**Figure 15:**
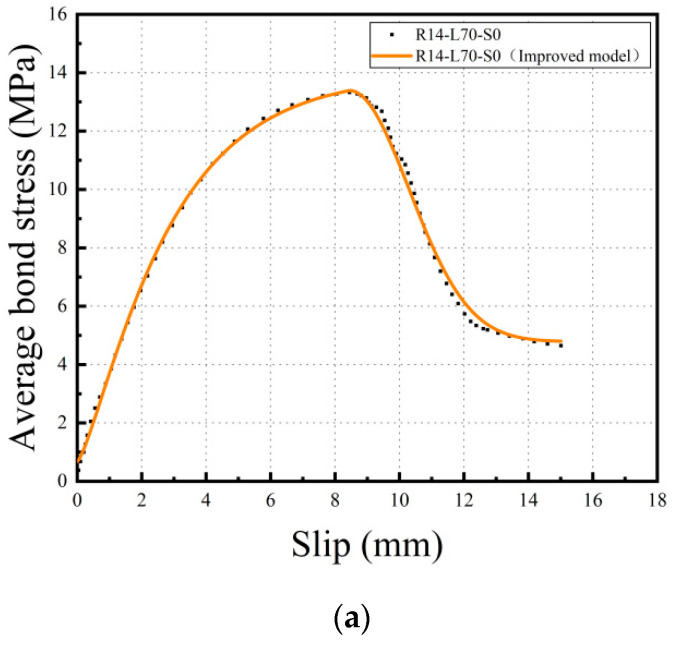

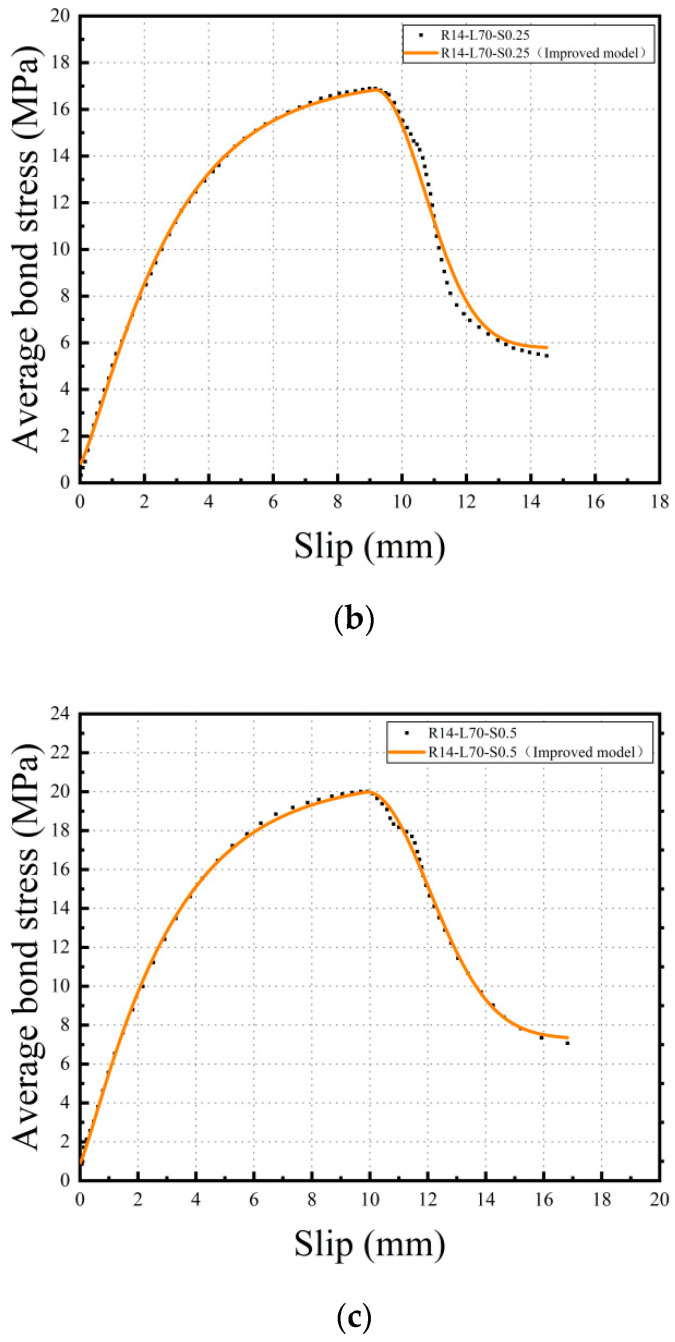
Comparison of experimental curve and improved model curve. (**a**) Comparison of R14-L70-S0 and improved model curve; (**b**) Comparison of R14-L70-S0.25 and improved model curve; (**c**) Comparison of R14-L70-S0.5 and improved model curve.

According to the aforementioned analysis, this paper proposes an improved bond-slip constitutive model between steel bars and the SMAF-ECC matrix, in which the rising section of the bond stress is based on the CMR model and the descending section is based on the Wu model, and a bond strength influence coefficient is introduced, as shown in Equation (7). The comparison between the fitting curve obtained using this improved bond-slip constitutive model and the experimental curve is shown in Figure 15, and the fitting parameters are listed in Table 7. As shown in Figure 15 and Table 7 that the improved bond-slip constitutive model has a good fitting effect with the experimental curve, and the R^2^ value reaches 0.99.

(7) $$\tau =\left\{ \begin{array}{ll} \psi_{\left(x\right)}{\left[1-\exp\left(-s/s_{r}\right)\right]}^{p} & \left(0\leq s\leq s_{u}\right)\\ \psi_{\left(x\right)}\left\{\tau_{0}/\psi_{\left(x\right)}+(1-\tau_{0}/\psi_{\left(x\right)})\exp\left[\beta {\left(s/s_{u}-1\right)}^{2}\right]\right\} & \left(s_{u}<s\leq s_{0}\right) \end{array}\right.$$

## 4. Conclusions

This study aims to explore the bond characteristics between steel bars and SMAF-ECC composite materials by conducting direct tensile tests on a total of 33 specimens. The primary focus of the analysis was to investigate the impact of various factors, including steel bar diameter, bond length, and SMAF volume fraction. Furthermore, based on the findings obtained from the experimental data, a bond-slip constitutive model was developed. The key findings and conclusions derived from this research are as follows:

1. The failure mode of the pull-out specimens is mainly steel bar pull-out failure, and no through-cracks appeared in the SMAF-ECC matrix when the specimens failed, indicating good tensile capacity of the matrix; the specimens with SMAF have significantly improved bond strength compared with those without SMAF, with the specimen of steel bar diameter 12 mm, bond length 70 mm, and SMAF volume fraction 0.5% showing the largest increase in bond strength, reaching 52.96%.
2. Within the scope of the experiment, it was found that as the diameter of the steel bar increased, the rib height relatively decreased, the rib spacing relatively increased, and the mechanical interlocking force decreased, resulting in a decrease in bond strength. When the bond length exceeded the effective bond length, an increase in bond length would lead to a smaller increase in tensile load than in bond area, a more uneven distribution of bond stress, and greater radial shrinkage of the reinforcing steel, thereby causing a decrease in bond strength. Increasing the volume fraction of SMAF effectively enhanced the crack resistance of the ECC matrix, as well as the shear and tensile strength of the matrix, thus improving the bond strength.
3. Based on the experimental data, the bond-slip curve between the steel rebar and SMAF-ECC matrix was obtained. An improved bond-slip constitutive model was proposed by introducing the SMAF fiber content influence coefficient to the existing model. Curve fitting analysis shows that the improved bond-slip constitutive model curve matches the experimentally obtained bond-slip curve well, with a determination coefficient of 0.99.

## Figures and Tables

**Table 1:** Weight composition ratio of the ECC material.

Cement /kg	Fly Ash /kg	Sand Cement Ratio	Water Cement Ratio	Water Reducing Agent/kg	Fiber Admixture * (%)
1.0	2.4	0.36	0.25	0.0082	2.0

* The fiber content is expressed as a percentage of the volume.

**Table 2:** Uniaxial tensile mechanical property index of SMA.

Diameter (mm)	Starting Point of Stress Platform		Ending Point of Stress Platform		Tensile Strength ^a^ (MPa)	Ultimate Strain ^b^ (%)
	Strain (%)	Stress (MPa)	Strain (%)	Stress (MPa)		
1.0	1.55	441.86	16.29	589.63	1111.01	25.53

^a^ The ultimate tensile strength is determined by extracting the peak stress from the stress-strain curve obtained through direct tensile testing of the SMA specimen. ^b^ The ultimate tensile strain is characterized as the strain at the fracture point observed in the stress-strain curve obtained through direct tensile testing of the SMA specimen.

**Table 3:** Mechanical characteristics of rebars.

Diameter (mm)	Yield Strength (MPa)	Tensile Strength (MPa)	Elastic Modulus (GPa)
12	470 ± 1	589 ± 2	182 ± 1
14	464 ± 1	579 ± 1	185 ± 1
16	471 ± 1	568 ± 2	181 ± 8

**Table 4:** Design of Tensile Test Scheme.

Specimen	Steel Bar Diameter	Bond Length	Volume Fraction of SMAF
R12-L70-S0	12 mm	70 mm	0%
R14-L70-S0	14 mm	70 mm	0%
R16-L70-S0	16 mm	70 mm	0%
R14-L50-S0.25	14 mm	50 mm	0.25%
R14-L90-S0.25	14 mm	90 mm	0.25%
R14-L70-S0.25	14 mm	70 mm	0.25%
R12-L70-S0.5	12 mm	70 mm	0.50%
R14-L70-S0.5	14 mm	70 mm	0.50%
R16-L70-S0.5	16 mm	70 mm	0.50%
R14-L50-S0.5	14 mm	50 mm	0.50%
R14-L90-S0.5	14 mm	90 mm	0.50%

Note: In the specimen code, R indicates the diameter of the steel bar, L denotes the length of the bond, and S signifies the volume fraction of SMAF.

**Table 5:** Test Results.

Specimen	Ultimate Load/N	Bond Strength/MPa	Failure Mode
R12-L70-S0	36,050.43	13.67	Pull Out Failure
R14-L70-S0	40,777.92	13.25	Pull Out Failure
R16-L70-S0	45,910.13	13.05	Pull Out Splitting Failure
R14-L50-S0.25	37,084.43	16.87	Pull Out Failure
R14-L90-S0.25	64,597.40	16.33	Pull Out Failure
R14-L70-S0.25	50,840.08	16.52	Pull Out Failure
R12-L70-S0.5	52,189.12	20.91	Pull Out Failure
R14-L70-S0.5	60,812.71	19.76	Pull Out Failure
R16-L70-S0.5	67,863.74	19.62	Pull Out Splitting Failure
R14-L50-S0.5	45,941.06	20.90	Pull Out Failure
R14-L90-S0.5	76,374.85	19.30	Pull Out Failure

**Table 6:** Parameter Fit.

Specimen	Wu Parameter			CMR Parameter		
	α	β	R^2^	Sr	p	R^2^
R14-L70-S0	0.535	−10.789	0.97	2.287	1.214	0.99
R14-L70-S0.25	0.493	−18.130	0.96	2.392	1.163	0.99
R14-L70-S0.5	0.503	−11.241	0.97	2.623	1.104	0.99

Note: R^2^ represents the determination coefficient.

**Table 7:** Modified Parameter Fit.

Specimen	Modified Model Parameter			
	Sr	p	β	R^2^
R14-L70-S0	2.417	1.368	−10.437	0.99
R14-L70-S0.25	2.440	1.313	−17.896	0.99
R14-L70-S0.5	2.795	1.209	−10.781	0.99

## Data Availability

Not applicable.
